# Conflicting demands on a modern healthcare service: Can Rawlsian justice provide a guiding philosophy for the NHS and other socialized health services?

**DOI:** 10.1111/bioe.12568

**Published:** 2019-03-18

**Authors:** Zoë Fritz, Caitríona Cox

**Affiliations:** ^1^ THIS institute Cambridge Biomedical Campus, Cambridge, U.K.; Division of Health Sciences Warwick Medical School University of Warwick Coventry U.K; ^2^ School of Clinical Medicine Cambridge University Cambridge U.K

**Keywords:** ethics, health services, physician–patient relations, policy, social justice

## Abstract

We explore whether a Rawlsian approach might provide a guiding philosophy for the development of a healthcare system, in particular with regard to resolving tensions between different groups within it. We argue that an approach developed from some of Rawls’ principles – using his ‘veil of ignorance’ and both the ‘difference’ and ‘just savings’ principles which it generates – provides a compelling basis for policy making around certain areas of conflict. We ask what policies might be made if those making them did not know if one was patient, doctor, nurse or manager – in this generation or the next.

We first offer a brief summary of Rawls’ approach and how we intend to extrapolate from it. We examine how this adapted Rawlsian framework could be applied to specific examples of conflict within healthcare; we demonstrate how this framework can be used to develop a healthcare service which is both sustainable (in its training and treatment of staff, and in encouraging research and innovation) and open (to protect the powers and opportunities of those using the health service). We conclude that while Rawls’ approach has previously been rejected as a means to address specific healthcare decisions, an adapted veil of ignorance can be a useful tool for the consideration of how a just health service should be constructed and sustained. Turning the theoretical into the practical (and combining Rawls’ thought experiment with Scanlonian contractarianism), managers, doctors, patients, carers and nurses could come together and debate conflicting issues behind a hypothetical veil.

## 1 INTRODUCTION

It is an old cliché that doctors do not make good patients. They are often reluctant to seek help; they worry overly about rare complications; and, unable to retain emotional neutrality, they interfere with decision making. Yet doctors do not only present challenges to their treating physicians: they also often find the experience of becoming a patient challenging themselves. Sometimes the needs of patients can only be fully appreciated after a doctor is forced to spend some time ‘on the other side’1Klitzman, R. (2008). *When doctors become patients*. Oxford, UK: Oxford University Press. Rawls’ veil of ignorance thought experiment asked actors to create a society not knowing what position they would play in it. It is often referred to, but also often dismissed as being of hypothetical use only: there are few circumstances where actors in society change roles. Healthcare, however, provides such a setting: doctors are forced to be patients; patients may train to be nurses; nurses become managers; managers are not exempt from experiencing the healthcare service as relatives of the sick.

One of the major problems that any healthcare system faces is that of ‘competing, and sometimes conflicting, demands …’.2Oldham, J. (2013). Reform reform: An essay by John Oldham. *British Medical Journal*,* 347*, f6716. Differing demands extend far past simply those of doctors vs. patients. Financial obligations must be balanced with clinical; the organization must be structured so that it supports those who work within it in a range of different positions and levels of seniority; the needs of individuals must be weighed against the needs of society; and, finally, in order for a healthcare service to be sustainable, the needs of current and future generations must be balanced.

The problem of resolving conflicts between groups with differing demands and priorities represents a significant challenge – not just in a healthcare system, but also more generally. Indeed, the rules which govern societies must be acceptable to a range of citizens with differing views. Many philosophers have grappled with constructing a set of principles which would lead to a ‘good’ society which is just to different users: John Rawls is perhaps foremost among them.

We explore whether a Rawlsian approach might provide a guiding philosophy for the development of a healthcare system, in particular with regard to resolving tensions between different groups within it. We argue that an approach developed from some of Rawls’ principles – using his ‘veil of ignorance’, and both the ‘difference’ and ‘just savings’ principles which it generates – provides a compelling basis for policy making around certain areas of conflict in the healthcare system. We ask what policies might be made if those making them did not know if one was patient, doctor, nurse or manager – in this generation or the next.

We first offer a brief summary of Rawls’ approach and how we intend to extrapolate from it. We examine how this adapted Rawlsian framework could be applied to the development of a healthcare service which is both sustainable and open. We will use specific examples of conflict within healthcare systems to demonstrate how we might use Rawls to address such conflicts and work towards fair solutions. We conclude that while Rawls’ approach has previously been rejected as a means to address specific healthcare decisions, an adapted veil of ignorance can be a useful tool for the consideration of how a just health service should be constructed and sustained.

## 2 RAWLS’ VEIL OF IGNORANCE

Behind the ‘veil’, people do not know what their place in society will be – they are ignorant as to their race, ethnicity, gender, income, wealth, natural endowments and religious/political convictions.3Rawls, J. (1971). *A theory of justice*. Cambridge, MA: Belknap Press of Harvard University Press. They are therefore equal and impartial parties, in what is termed the ‘original position’. Rawls puts forward this key question: what principles would ‘free and rational persons concerned to further their own interests’ accept in such an original position? The terms that rational (and reasonable) agents would agree to from behind the veil are, ‘by virtue of their agreement alone’, just, and lead to equitability.4Sandel, M. J. (2009). *Justice: What's the right thing to do?* (1st ed.). New York, NY: Farrar, Straus and Giroux. Rawls uses the phrase ‘justice as fairness’, to reflect this principle: it ‘conveys the idea that the principles of justice are agreed to in an initial situation that is fair’.

Rawls argues that we can derive two main principles of justice that would be chosen:

1. All persons should have equal basic liberties.
2. Social and economic inequalities [of primary goods] need to satisfy two conditions:
  - They should be attached to positions/offices open to all under fair equality of opportunity;
  - They should be of greatest benefit to the least advantaged in society (known as the difference principle).5Rawls, *op. cit*. note 3.

‘Primary goods’ are defined (in Rawls’ second edition of *A theory of justice*) as ‘what persons need in their status as free and equal citizens, and as normal and fully cooperating members of society over a complete life’.6Rawls, J. (1999). *A theory of justice* (Revised edn.). Cambridge, MA: Belknap Press of Harvard Univeristy Press.


The difference principle postulates that primary goods should be distributed equally, unless an unequal distribution would be to everyone's – and in particular the worse‐off's – advantage. Economic inequalities are thus permissible only if they benefit to the least advantaged in society. Accordingly, some people may justifiably to be paid better than others as an incentive, provided that the incentives are needed to improve the welfare of the less well‐off. Rawls ultimately argues that rational and self‐interested people in the original position would agree to rules which maximize the minimum level of primary goods that they might find themselves with beyond the veil.

The question of whether ‘opportunity’ as a social good extends to healthcare has been debated. Norman Daniels emphasizes the relationship between normal functioning and opportunity: ‘Impairments of normal functioning, including early death, reduce the range of opportunity open to individuals in which they may construct or pursue “plans of life” … disease and disability thus diminish individual fair shares of the normal opportunity range.’7Daniels, N. (1985). *Just health care*. Cambridge, UK: Cambridge University Press. By extension, then, healthcare can be linked to opportunity, which is considered a primary good.

While the definition of what constitutes a primary good has been discussed, by Daniels and others,8Ekmekci, P. E., & Arda, B. (2015). Enhancing John Rawls's theory of justice to cover health and social determinants of health. *Acta Bioethica*,* 21*, 227–236 this paper does not set out to contribute further to this debate, nor indeed does it set out to add to the literature on the analysis or interpretation of Rawls’ extensive body of work.9Kukathas, C., & Pettit, P. (1990). *Rawls: A theory of justice and its critics*. Key contemporary thinkers. Cambridge, UK: Polity. Rather we propose building on Rawls’ basic framework, to establish how differing demands and competing interests within a healthcare system might be addressed justly.

It is worth noting that, whilst Rawls conceptualized a situation in which you cannot change between gender, or be rich and poor at the same time, in healthcare there is fluidity between roles. People can be in two positions simultaneously – patient and doctor, nurse and manager – and can move between the two. This tool can therefore be used more empathically (and therefore more realistically) than in other circumstances. Rawls’ approach is thus particularly useful when considering the health service, as it is not just a hypothetical construct, but one which we – as practitioners and patients – may experience in the flesh.

## 3 THE IMMEDIATE VS. FUTURE NEEDS OF PEOPLES: CREATING A SUSTAINABLE HEALTHCARE SERVICE

A healthcare system must balance the needs of those alive today, and those who will need to make use of its services in the future. This requirement – to fairly consider both current and future users – represents a significant challenge for policy makers. Although at first glance the principle that the patient should be at the centre of any health care system seems relatively uncontroversial, on closer examination it presents some problems. Is ‘the patient’ in question a current user of the healthcare service? We might assume that references to ‘patients’ encompass future as well as present users of health services – yet this becomes problematic when the needs of current and future persons are at odds. To provide a sustainable healthcare service, the needs of current patients may have to be compromised in order to meet fairly the needs of future patients.

Here we will first outline how a Rawlsian framework might be utilized to address issues surrounding sustainability in general. We will then briefly present some areas of potential conflict regarding the establishment of a health service which is both acceptable to current users and sustainable over time, using these examples to illustrate how a Rawlsian framework might be applicable.

### 3.1 Rawls and sustainability: General principles

Rawls addresses the issue of sustainability in his discussion of how just institutions should be maintained across time. The ultimate aim for Rawls is to produce a ‘steady‐state phase whereby just institutions are preserved from one generation to the next’.10Welburn, D. (2013). Rawls, the well‐ordered society and intergenerational justice. *Politics*,* 33*, 56–65. To achieve this, he proposes a ‘just savings’ principle, which is ‘an understanding between generations to carry their fair share of the burden of realizing and preserving a just society’.11Rawls, *op. cit*. note 3. Intergenerational responsibilities are considered within the framework of a ‘present entry of time’ interpretation of the original position.12Rawls, J. (1993). *Political liberalism*. New York, NY: Columbia University Press. In this version, people behind the veil of ignorance are still self‐interested contemporaries, but are now also ignorant of the position that their own generation holds in the timeline of generations. Bearing this in mind, an individual behind the veil of ignorance would not choose a system in which early generations are able to use resources – for example, fossil fuels – at an unsustainable rate. This is to protect oneself from the possibility of being in a much later generation which would then suffer from the lack of available resources (in this example, a generation with no access to fossil fuels). Yet Rawls also argues that, just as no generation has any stronger claim to advantage over any other, no one generation should bear an unreasonable burden in the creation and maintenance of just institutions. To return to the fossil fuels example, whilst early generations should not use fossil fuels at an unsustainable rate, they should also not be forced to be so frugal to not be able to make use of them at all. We will see how this applies to both the training of doctors and to working conditions and pay later in the paper.

The difference principle is also of some relevance in considering how the needs of current and future users of a healthcare system might be balanced. Rawls argues that people in the original position would agree to rules which maximize the minimum level of primary goods that they might find themselves with beyond the veil. People would thus aim to maximize the minimum amount/quality of healthcare they might receive. As we will explore with some examples below, such a system would often be inconsistent with the just savings principle, according to which people have a duty to make possible ‘the conditions needed to establish and to preserve a just basic structure over time’.13Rawls, J., & Kelly, E. (2001). *Justice as fairness: A restatement*. Cambridge, MA: Harvard University Press. The just savings principle thus constrains the difference principle: it would not permit one generation to distribute primary goods in such a way that the existing least well‐off had the maximum primary goods possible at the cost of future least well‐off people. The difference principle determines distributive justice *within* one generation, while the just savings principle could be applied *between* generations to safeguard the existence of just institutions in the future.

The question of how much current generations have an obligation to save for future generations has been addressed by Rawls using a two‐stage theory of intergenerational justice, comprising of an accumulation phase and a steady‐state phase. During the accumulation phase, people have an obligation to save as much as is necessary for future generations to reach a sufficientarian threshold, which Rawls defines as ‘the conditions needed to establish and to preserve a just basic structure over time’.^13^ Once just institutions have been established, a steady state is reached, in which people have an obligation merely to leave as much for the next generation as they inherited. Rawls is explicit in his claim that once this steady state has been reached, there is no obligation for people to save in order to accumulate capital: ‘I follow Mill's view that the purpose of saving is to make possible a just basic structure of society; once that is safely secured, real saving (net increase in real capital) may no longer be necessary’.14Rawls, J. (1999). *The law of peoples: With ‘The idea of public reason revisited’*. Cambridge, MA: Harvard University Press.


It is worth noting that several philosophers have suggested that a just system could be achieved contemporaneously by debating principles no one could ‘reasonably reject’, rather than a (hypothetical) prospective agreement of principles that everyone would agree to. Scanlonian contractualism argues that individuals or groups (in this case doctors or nurses or patients, although Scanlon did not explicitly consider healthcare) should pursue only interests they could justify to others who have their own interests to pursue, and would be motivated by ‘the desire for reasonable agreement’.15Scanlon, T. (2003). *The difficulty of tolerance: Essays in political philosophy*. Cambridge, UK: Cambridge University Press. Habermas’ view that the human potential for reason can lead to a more just society supports this as an achievable goal.16Habermas, J. (1989). *The structural transformation of the public sphere: An inquiry into a category of bourgeois society*. Cambridge, MA: MIT Press. Norman Daniels addressed the needs of priority setting and rationing of healthcare resources with the development of an ‘accountability for reasonableness’.17Daniels, N., & Sabin, J. E. (2008). Accountability for reasonableness: An update. *British Medical Journal*,* 337*, a1850; Daniels, N. (2008). *Just health: Meeting health needs fairly*. Cambridge, UK: Cambridge University Press. To hold decision makers accountable for the reasonableness of their decisions, Daniels argued that ‘the process must be public (fully transparent) about the grounds for its decisions; the decision must rest on reasons that stakeholders can agree are relevant; decisions should be revisable in light of new evidence and arguments; and there should be assurance through enforcement that these conditions (publicity, relevance, and revisability) are met.’ His work drew on a qualitative study by Singer et al., who examined how priority setting was decided upon in two healthcare situations.18Singer, P. A., Martin, D. K., Giacomini, M., & Purdy, L. (2000). Priority setting for new technologies in medicine: qualitative case study. *British Medical Journal*,* 321*, 1316–1318. Multiple stakeholder perspectives were represented, and the emphasis was on consensus building and transparency (and justification) of the rational for decisions made.

Arguably having multiple (reasonable) stakeholders reaching a consensus is a practical way of applying Scanlonian contractualism, but it addresses only the views and needs of those currently using or working in the service – not of future generations. As Kumar has argued, future generations can be harmed if the ‘wrongdoer’ fails to ‘live up to her responsibilities with respect to the wronged’.19Kumar, R. (2003). Who can be wronged? *Philosophy and Public Affairs*,* 31*, 99–118. To not consider future generations in planning responses to current day health service crises is to neglect our responsibilities to future generations of health service users.

What more might applying a Rawlsian framework add to the approaches of consensus building and ‘accountability of reasonableness’? Practically speaking, the increasing inclusion of ‘patient and public involvement’ (PPI) in decision making about research agendas and healthcare delivery reflects well upon the institutions’ recognition of the importance of multiple perspectives. One issue with this approach is that any individual group can only represent their current interests. In addition, these interests are framed within current social biases. A Rawlsian model which includes an intergenerational perspective requires us to undertake an exercise in current and future empathy, enabling us to design a system that will be good for the current us – in whatever state we are in – and the future us. Since it takes us into a hypothetical realm, we are also freed from pragmatic limitations of how things currently work and can imagine what an ideal system might look like, if we were designing it unaware of whether we would be nurse, doctor, manager, policy maker or patient – current or future.

We thus present Rawls’ work as a more useful intellectual framework. Not only can Rawlsian justice be of use in determining how conflicting demands within generations might be resolved, but, extended, it allows us to balance conflicts across generations. Such an approach can help us to develop the structure of a health service, which is not only just to all current users, but sustainable – and thus just to future users also.

### 3.2 Applying these principles to some examples of conflicts

We will now examine a few examples of conflicts which exist between current and future users of a health service: training programmes for doctors, working conditions and pay, and the need for innovation/research. In all these examples, we argue that the principles set out in the above section can be of considerable use in attempting to resolve these conflicts.

#### 3.2.1 Training of staff

A tension exists between the needs of trainees and the needs of patients. A (clichéd but useful) example is that of the trainee surgeon. To improve their skills – and ultimately their ability to provide good‐quality care to patients – trainees must operate on patients. A trainee will be less skilled than a more senior surgeon in performing a certain procedure: ‘no matter how many protections are in place, on average these cases go less well with the novice than with someone more experienced’.20Gawande, A. (2002, Jan 28th). The learning curve. *The New Yorker*, 52–61. If we were to consider only the needs of the current patient, operations should be performed only by the most skilled practitioner available. Yet, if only the most experienced surgeon available operated on patients, then junior trainees would not have the opportunity to become sufficiently skilled to take over on their senior's retirement – a clearly unsustainable model. The just savings principle provides a compelling basis for the need to train new surgeons adequately, through a consideration of what the current generation owes to future generations.

Within this framework, we might agree to a system in which trainees are allowed to operate on patients, but with adequate supervision, and having had the best possible training prior to operating on patients (through the use of simulations or models). We might further stipulate that all patients have an equal chance of being operated on by a trainee: we would not, for example, consent to a system in which the wealthy are able to pay more to ensure a more experienced surgeon.21Arguably this does happen in the UK already: individuals can elect to have some operations done privately, and private work is only conducted by experienced surgeons. This option, however, is only available for elective, routine surgery. This approach would ensure that the needs of the current surgeons and patients were not satisfied at the expense of future ones.

#### 3.2.2 Working conditions and pay

Issues regarding working conditions and pay also represent a challenge for the development of a sustainable healthcare system. In light of recent disputes over junior doctor contracts22Toynbee, M., Al‐Diwani, A. A., Clacey, J., & Broome, M. R. (2016). Should junior doctors strike? *Journal of Medical Ethics*,* 42*, 167–170. and student nurse bursaries,23Glasper, A. (2016). Will the end of bursaries lower student nurse applications to UK universities? *British Journal of Nursing*,* 25*, 566–567. the issue of how a healthcare system should not only train but *treat* its employees is of particular relevance: how should the needs of staff should be balanced with the needs of patients?

Consider how you might approach the issue of working conditions from behind the veil of ignorance, not knowing whether you will be a patient, a nurse, a doctor, or a manager (and remembering that you could be more than one within a lifetime). As a patient, your main priority would be receiving appropriate attention from well trained staff, whilst as an employee you would be likely to place greater emphasis on ensuring reasonable working conditions within which you could provide care. A manager would have a greater interest in designing a system in which staff are utilized in the most cost‐effective manner.

We can justify reasonable working conditions for employees using a Rawlsian approach from two separate perspectives. Evidently, good working conditions would be favourable for the employees themselves. If we assume that there is a possibility that people will end up working within the healthcare system, then it is reasonable to argue that from behind the veil of ignorance people would agree to a basic structure which ensures acceptable working conditions. To take the example to an extreme, people would not design a healthcare system which is run by slaves, even if this would provide the most cost‐efficient service for the majority of the population; they would want to protect themselves against the unfortunate possibility of ending up as one of the said slaves. The other important justification for ensuing reasonable working conditions is to create a sustainable workforce. As discussed above, Rawls’ just savings principle provides a basis for the need for a sustainable healthcare system, thus necessitating working conditions into which new staff can be recruited and retained.

The issue of how much healthcare providers should be paid is a complex topic, as the amount staff are paid must be balanced against the cost to patients of reducing the amount of available resources for treatments. Rawls provides guidance on how income should be distributed through the difference principle. Using this principle, it is acceptable to pay doctors (and others who work in health care) more than the average salary in order to entice talented individuals into the profession, so as to secure their services to provide medical care for other less well‐off members of society. It is important to note that such higher salaries for doctors would not reflect their being intrinsically deserving of such wealth on account of their talents or efforts, or of having any ‘privileged moral claim to the fruits of their labour’.24Rawls, *op. cit*. note 3. Rather, using a Rawlsian approach to explore what staff should be paid involves primarily considering how much remuneration is required for recruitment and retention, something explicitly mentioned by the Doctors’ and Dentists’ Review Body (DDRB) in the consideration of ‘the need to recruit, retain and motivate doctors and dentists’.25B.L. Amy R, Butler M, et al. (2016). Review body on doctors’ and dentists’ remuneration: Forty‐fourth report D.o.H.a.O.o.M. Economics, ed. London, UK: Review Body on Doctors' and Dentists' Remuneration, Department of Health and Social Care and Office of Manpower Economics. We accept that determining exactly how much different healthcare professionals should be paid to reflect this is challenging – a full discussion of the issue is beyond the scope of this paper. We rather highlight how using Rawls’ framework can help address issues relating to both working conditions and pay for employees within a healthcare system. Ultimately, both must be set at a level which is acceptable to employees, in order to recruit and retain staff so as to ensure a sustainable workforce.

#### 3.2.3 Innovation and research

Finally, consider the need for to strive for improvement and innovation within a healthcare system. It seems clear that in order to provide the best care possible for future generations, a health service must embrace innovation and actively promote research. A static healthcare service would not be (or at least, long remain) an excellent one. At times, the demands of engaging in research can be at odds with the needs of current users of a healthcare service: consider the investment of money into laboratory‐based research as opposed to treatments which might provide an immediate benefit.

One could argue that although Rawls’ just savings principle can be used to justify the need for a sustainable healthcare system, it cannot be convincingly applied to justify the need for improvement and innovation. After all, once the steady state has been reached, people do not need to accumulate savings for future generations. If applied to a healthcare service, arguably we do not have any obligation to use resources to improve services for future users, so long as we leave them with a service which is as good one we received. As Gaspart and Gosseries note, ‘allowing our generation to continue to save once the sufficiency level has been reached is unfair … towards the least well‐off within our generation’.26Gaspart, F., & Gosseries, A. (2007). Are generational savings unjust? *Politics, Philosophy & Economics*,* 6*, 193–217.


Such an argument is flawed on two accounts. Firstly, it assumes that the accumulation of knowledge can be considered in the same way that the accumulation of savings can be. This is problematic, as accumulating knowledge in one generation does not necessarily deprive the current generation of opportunities in the same way that saving money does. Indeed, Gaspart and Gosseries concede that in some instances in which the ‘growth that does not hurt the current generation’, savings may be allowed even in the steady state. They use the example of a fisherman teaching his offspring to fish, noting that ‘experience naturally accumulates from one generation to another, at no extra cost for the present generation’.^25^ This could be applied in some cases to the advancement of medical knowledge; if, for example, doctors note that treatment x is superior to treatment y in the management of a given condition, it would not be unjust for the medical profession to pass on this knowledge to the next generation.

A second – perhaps more critical – question is whether we can really claim to have reached a ‘steady state’ in the health service. Rawls describes the steady state as a situation in which ‘just institutions are firmly established and all the basic liberties effectively realised’.27Rawls & Kelly, *op. cit*. note 13. We concede that it is difficult to neatly apply these conditions – originally intended to be applied to just institutions – to a healthcare service. Indeed, defining what constitutes a ‘sufficiency level’ of medical knowledge is challenging: in the context of medical research, it is unclear exactly what the ‘steady state’ would look like, or indeed if it could ever truly be reached. If we assume that the healthcare service is still in the accumulation phase, we can justify the need for research and innovation in order to improve the service provided to future generations. Such an approach can more firmly ground the requirement for improvement within a health service, even if there is some cost to current generations.

## 4 POWERS AND OPPORTUNITIES: GOVERNMENT VS. DOCTORS VS. PATIENTS – CREATING AN OPEN AND ACCOUNTABLE SYSTEM

In Section 3, we have examined specific areas of sustainability. We now turn to the overall structure and governance of a health service – we are writing from the perspective of doctors working in the NHS, but the principles could be applied to any socialized healthcare system. Since *power* and *opportunities* are primary goods, it follows that individuals should have the opportunity to exert some power over the system which provides their healthcare. To do so, the system must be open and accountable: open in terms of its transparency of decision making to patients and staff; accountable in terms of the individuals and organizations which make decisions about the running of the health service. In most democratic countries this accountability is wrapped up in the general accountability of the national electorate, who are balancing their work, expertise and decision making in healthcare with a myriad other demands. When the election cycle comes up they can be voted back in or out based on their performance in any of those domains.

As a result, the accountability to decision making in healthcare policy at the highest level is not very direct, nor is it responsive. As an example, when the Health and Social Care Act bill28Health and Social Care Act (2012). Available at: http://www.legislation.gov.uk/ukpga/2012/7/contents/enacted. Accessed Dec 11, 2018. was introduced in 2012, it was vociferously objected to by most professional bodies,>29Chan, J., Currie, J., Pool, E., Ross, C., Ward, E.; Big Society NHS. (2011). Royal Colleges must act over Health and Social Care Bill. *Lancet*,* 378*, 1543; Davies, E. (2012). BMA meeting: BMA representatives back calls to repeal Health and Social Care Act. *British Medical Journal*,* 344*, e4395. who found themselves nevertheless powerless to change the proposals, which were passed by the elected government. In addition, the issue of financing healthcare has become increasingly challenging. It is recognized that an aging population and increasing abilities to treat a variety of disease lead to rising costs; yet the election cycle discourages increases in taxation.

The question therefore is, if the funding and management of a socialized healthcare system were being constructed behind the veil of ignorance, what would be different? It is likely that individuals would choose policies which allowed the users and staff greater powers and opportunities than are currently available to them.

### 4.1 Financing a healthcare system

If we did not know whether we were the current or future patients or staff, any financial planning would be likely to take into account the need for the institution to be adequately financed in the future: we would not suggest unsustainable expenses, and we might suggest a system of financing which was sustainable outside the realms of political party control.

There are many possible methods of financing that have been discussed. Those which would disadvantage the poor (for example a small fixed fee for attending a primary care physician or family doctor)30Commission on the Future of Health and Social Care in England (2014). *A new settlement for health and social care: Final report*. London, UK: The Kings Fund. would be rejected in a Rawlsian approach. A method which placed short‐term benefits ahead of long‐term planning (for example the private finance initiative for new hospitals)31Dunnigan, M. G., & Pollock, A. M. (2003). Downsizing of acute inpatient beds associated with private finance initiative: Scotland's case study. *British Medical Journal*,* 326*, 905; Pollock, A. M., & Price, D. (2010). The private finance initiative: The gift that goes on taking. *British Medical Journal*,* 341*, c7175 would be rejected on the grounds of wrongdoing to future generations, but a hypothecated tax might not be.32Layard, R., & Appleby, J. (2017). Is it time for a dedicated tax to fund the NHS? *British Medical Journal*,* 356*, j471. As a population we consistently report valuing a healthcare system which is free on the point of delivery,33Robertson, R. (2017). *Public satisfaction with the NHS and social care in 2016*. London, UK: The Kings Fund. so ‘ring‐fencing’ the taxes for this service might be palatable. A full discussion of the different approaches to financing the NHS is beyond the scope of this paper, however a Rawlsian approach provides a good framework for this debate.

### 4.2 Health policy

The lack of responsiveness and direct accountability to the users of the NHS has meant that changes to policy have often been responsive to immediate challenges rather than proactively planning for the long term. Investment in interventions whose positive effects will only be seen in 15 years has little attraction for those elected on 5‐year terms, and there is insufficient time to pilot changes in a few selected areas before implementing them nationally. This problem is not restricted to the NHS, and lessons can be learnt from its history. A Rawlsian approach, taking into account the just savings principle, would be unlikely to result in such a system. Along with the segregated finances might also come a secondary (elected?) body of experts to advise on policy decisions. If you were unsure whether you would be a present or future individual you would be more likely to invest in preventative medicine and policies which were well tested. In addition, the opportunity to challenge recommendations, or to have more open consultations about policy changes and targets might be valued.

A recent court case in clinical practice in the UK emphasized that patients had the right to know what decisions were being made about them in order that they could have the opportunity to ask questions and ask for a second opinion.34R (Tracey) vs. Cambridge University Hospital NHS Foundation Trust and others [2014] EWCA Civ 822. Extrapolating this to broader clinical practice, patients and clinicians should have the opportunity to challenge policies that are being made that will directly affect the delivery or receipt of healthcare, and to ask for expert opinions on what the best policy might be.35Petit, P. (2008) Three conceptions of democratic control. *Constellations*,* 15*, 46‐55; Petit, P. (2006). Rationality, reasoning and group agency. *Dialectica*,* 61*, 495–519. Clinicians and patients might have questioned, for example, the ‘target’ of not being in the emergency department for more than 4 hours which has driven much of the activity in acute trusts in the UK over the last 10 years. It was not piloted, and has been shown to have created inefficiencies with unnecessary patient moves and inappropriate triaging of patients, while not reducing mortality.36Boyle, A., & Mason, S. (2014). What has the 4‐hour access standard achieved? *British Journal of Hospital Medicine*,* 75*, 620–622.


The current system does not afford sufficient powers or opportunities to those who work in and use the NHS or other similar health systems: they are often not able to challenge or influence policies which directly impact upon them. A system designed behind a veil of ignorance might allow those that work within the health service to influence it better.

### 4.3 Powers and opportunities on a micro level

In addition to the lack of power in terms of organizational change or management, many of the basic interactions that take place within a health service – between doctors and patients, between nurses and doctors and between managers and their staff – are imbalanced, without the opportunity to challenge them.

Consent to surgery, disclosure about poor prognoses, informing patients about resuscitation decisions, have all been areas of practice which went unchallenged for decades; change has come about in response to pressure groups, research or legal case. Sometimes the response has been to correct the previous injustice and create a new one: in the United States, where patient power extends to requesting treatments, physicians report that they are delivering aggressive and invasive treatments at the end of life that they themselves would not want.37Periyakoil, V. S., Neri, E., Fong, A., & Kraemer, H. (2014). Do unto others: Doctors’ personal end‐of‐life resuscitation preferences and their attitudes toward advance directives. *PLoS One*,* 9*, e98246. If a social contract were being drawn up afresh, we might be forced to question some of the practices we all take for granted, and proactively design a better system. Behind the ‘veil of ignorance’ would people design a system which left those treating or being treated so impotent?

Consider how one would approach some of the clinical examples above from behind the veil of ignorance. Historically, doctors would often withhold information – particularly about poor prognoses – from their patients. From the original position, one would be unlikely to design a system in which doctors have no duty to disclose such information, due to the potential of being a patient in such a system – uninformed about one's own condition and unable to properly engage in discussion about it. One would equally not wish for a system in which potentially distressing information was given indiscriminately to patients, as one might be a patient who has a strong personal preference for not knowing the details of a condition. Considering the doctor's perspective from behind the veil, a system would need to leave room for a doctor to employ some clinical judgement in deciding what information to disclose and how to disclose it: few doctors would want to be in a system where they are strictly obligated to explain, for example, every mildly deranged blood test or every possible (but unlikely) cause for the patient's symptoms. Such a standard would be time‐consuming and impractical, and would thus be rejected. Using the veil of ignorance to examine how information should be shared both from the patient and doctor perspective, we might reach a system in which patients guide how much information they want about their prognosis, with doctors using clinical judgement to disclose this information in the most appropriate way. The same process can be applied to the other information‐sharing examples mentioned above, such as consenting to surgery and discussing resuscitation decisions. A Rawlsian approach permits us to examine the conflicting demands that different groups have when considering how power should be balanced between them.

As demonstrated above, the tool of the original position could be helpful in examining how powers and opportunities are distributed between individuals within a healthcare system. Not knowing if you would be patient or doctor, it is likely that a system would be created which balanced patient need for openness and autonomy with healthcare professionals’ need for discretion and judgement in what and when information is communicated.

Thus openness – in the leadership, governance, and day‐to day workings of the NHS – would lead to better distribution of power and opportunity, and contribute to the sustainability of the NHS as a structure.

## 5 CONCLUSION: CAN RAWLS PROVIDE A GUIDING PHILOSOPHY FOR THE NHS?

While Rawls has been rejected as being useful for specific healthcare decisions,38Shevory, T. C. (1986). Applying Rawls to medical cases: An investigation into the usages of analytical philosophy. *Journal of Health Politics, Policy & Law*,* 10*, 749–764. we have presented examples of how a Rawlsian approach could be used to decide how a health service should be constructed and run. It provides a useful framework for the examination – and, arguably, the just resolution – of many conflicts.

The basic structure of a healthcare system agreed to by people in the original position (including the intergenerational caveat) would bear similarities to the NHS as it currently exists – for example, Bevan's initial requirements of universal access and treatment based on clinical need (not ability to pay).39U.K. Parliament. (1946). The National Health Service Act. It would not, however, be identical. Through the application of an adapted Rawlsian analysis we argue that two further general principles emerge, which might provide a normative basis for the principles which would best guide a just healthcare system.

First, in considering a variety of examples in which the immediate needs of individuals conflict with the future needs, it is clear that there is a strong requirement for the development of just institutions which are sustainable. Working conditions should promote adequate recruitment, retention and training of staff; the financial and structural infrastructures should include long‐term considerations. Second, increased openness, to protect the power and opportunities of both those who work in the healthcare service and those who receive care, should be prioritized.

We have provided our own theories on what a health service might look like if it were designed by a group behind an intergenerational veil of ignorance. The complexity of striking the balance between interests might be elicited by asking a group of managers, doctors, patients, carers and nurses to come together and debate these issues behind a hypothetical veil, thus combining Rawls’ thought experiment with Scanlonian contractarianism. This approach could be first investigated with qualitative research, and ultimately translated into practice.

There are an increasing number of challenges facing the national health service on its 70^th^ birthday, and many other healthcare systems around the world. We argue that a philosophical framework is required to address them: Rawls’ theory of justice provides a guiding moral philosophy which can offer a basis for the resolution of many of the conflicts which health services face.

## CONFLICT OF INTEREST

The authors declare no conflict of intrest.

